# Pharmacological management of tauopathies

**DOI:** 10.1002/alz70857_103676

**Published:** 2025-12-25

**Authors:** Raul Medina Rioja, Alonso Morales‐Rivero, Indira Ruth Garcia Cordero, Gabor G. Kovacs, Carmela Tartaglia, Blas Couto

**Affiliations:** ^1^ Instituto Nacional de Neurología y Neurocirugía “Manuel Velasco Suárez”, Mexico, DF, Mexico; ^2^ Toronto Western Hospital, University Health Network Memory Clinic, Toronto, ON, Canada; ^3^ Universidad de San Andres, Victoria, Buenos Aires, Argentina; ^4^ Tanz Centre for Research in Neurodegenerative Diseases, University of Toronto, Toronto, ON, Canada; ^5^ Rossy PSP Program, University Health Network and the University of Toronto, Toronto, ON, Canada; ^6^ Laboratory of Progressive Neuromotor and Neurobehavioral Disorders at Instituto de Neurociencia Cognitiva y Traslacional (INCYT), CABA, Buenos Aires, Argentina; ^7^ Unidad de Movimientos Anormales, Institute of Neuroscience of the Fundacion Favaloro, CABA, Buenos Aires, Argentina

## Abstract

Tauopathies, a group of neurodegenerative disorders characterized by tau protein aggregation, encompass diseases such as progressive supranuclear palsy, and corticobasal degeneration. While the development of disease‐modifying therapies remains a primary research focus, current pharmacological interventions are predominantly directed towards symptomatic management. This review aims to summarize the evidence‐based approaches for addressing the motor, autonomic, and systemic manifestations of tauopathies, highlighting both their therapeutic benefits and limitations. For motor dysfunction, dopaminergic agents and muscle relaxants are explored, though their benefits in PSP and CBS are often modest. Sleep disturbances and autonomic dysfunction, which are prevalent but underrecognized in tauopathies, can be managed using targeted pharmacological strategies, with emerging evidence supporting the use of melatonin and other agents. We will discuss the current significant gaps in optimizing these treatments to meet the complex needs of patients and their family. The potential for repurposing existing drugs and the role of novel symptomatic therapies is currently under investigation. By providing an updated synthesis of pharmacological management strategies, clinicians attending this session will get the evidence‐based, patient‐centered care skills for treatment of PSP and CBS.

